# Leveraging the Intersection between Age-friendly Universities and Age-Friendly Communities

**DOI:** 10.1093/geroni/igab046.377

**Published:** 2021-12-17

**Authors:** Kathy Black

**Affiliations:** University of South Florida, Sarasota-Manatee, Florida, United States

## Abstract

Age-friendly Universities represent a growing contribution to the worldwide age-friendly movement. For universities, the international effort aims to highlight the role higher education plays in responding to the opportunities associated with an aging population. The initiative outlines ten principles to engage older adults via collegiate mission pertaining to research, education and service. Shared practices suggest diverse and unique application of the guiding tenets across participating colleges and universities. However Age-friendly Universities are also part of a broader ecosystem, situated in geographic locales reflecting actual or prospective age-friendly community status. The global Age-friendly Community movement is a decade-old effort to improve the environments in which we age via a cyclical process. This paper identifies the intersection between Age-friendly University principles and Age-Friendly Community processes and discusses reciprocal considerations for mutual advancement of the broader movement.

